# Exercise intervention for children with acute leukemia: a best evidence summary

**DOI:** 10.3389/fped.2026.1931097

**Published:** 2026-09-17

**Authors:** Wei Zhou, Long Li, Jingjing Ma, Junxia Zhou

**Affiliations:** 1 Department of Pediatric Hematology, West China Second University Hospital, Sichuan University, Chengdu, China; 2 Key Laboratory of Birth Defects and Related Diseases of Women and Children (Sichuan University), Ministry of Education, Chengdu, China

**Keywords:** acute leukemia, children, evidence-based nursing, exercise intervention, review

## Abstract

**Background:**

Acute leukemia (AL) is the most common malignancy in childhood. Despite significant improvements in survival rates, long-term high-intensity chemotherapy often leads to severe physical dysfunction and cancer-related fatigue (CRF) in pediatric patients. Although exercise intervention is a significant non-pharmacological physical therapy for improving patient prognosis, clinical practice still lacks unified, evidence-based standardized guidance.

**Objective:**

To systematically retrieve, evaluate, and synthesize evidence regarding exercise interventions for children with AL, providing a decision-making reference for multidisciplinary clinical teams to develop standardized and individualized exercise rehabilitation programs.

**Methods:**

Guided by the Joanna Briggs Institute (JBI) evidence-based practice framework, a comprehensive search was conducted in domestic and international databases, including UpToDate, BMJ Best Practice, JBI, PubMed, Cochrane Library, CNKI, and Wanfang, to identify clinical decision supports, guidelines, expert consensuses, systematic reviews, and meta-analyses related to exercise interventions for pediatric AL. The search period spanned from database inception to January 2026. Methodological quality appraisal and data extraction were performed independently by two researchers.

**Results:**

Eighteen publications were included, consisting of 2 clinical practice guidelines and 16 systematic reviews. Seventeen items of best evidence were extracted, covering eight dimensions: intervention principles, safety, exercise modalities, intensity, frequency and timing, stage-specific strategies, intervention settings, and outcome improvement.

**Conclusion:**

Children with acute leukemia should engage in long-term, individualized, multimodal exercise interventions under professional supervision. Healthcare professionals should dynamically adjust exercise protocols based on the specific treatment phase. While ensuring safety, incorporating game-based formats can enhance patient adherence, thereby effectively improving bone mineral density, cardiorespiratory endurance, and quality of life.

**Systematic Review Registration:**

https://www.crd.york.ac.uk/prospero/view/CRD420261341113/1/0, PROSPERO CRD420261341113.

## 1 Introduction

Acute leukemia (AL) is the most prevalent malignancy in childhood (1), with acute lymphoblastic leukemia (ALL) exhibiting the highest incidence, accounting for approximately 70% of all pediatric leukemia cases (2). In recent years, continuous advancements in medical technology and the implementation of multicenter clinical trials have significantly enhanced the diagnosis and treatment of pediatric AL, with the 5-year survival rate for ALL reaching 80%–90% (2). Consequently, the focus of AL research has shifted from solely improving survival rates to maintaining and restoring physical activity levels, enhancing long-term quality of life, and facilitating successful reintegration into society (3). Despite substantial improvements in survival outcomes, long-term high-intensity treatments, primarily chemotherapy, impose a heavy physiological burden on children. Prolonged pharmacological therapy and prolonged bed rest during hospitalization frequently lead to organ dysfunction, obesity, osteoporosis, and severe cancer-related fatigue (CRF), resulting in significant physical mobility impairments (4).

Studies indicate that children with leukemia undergoing induction or consolidation chemotherapy exhibit poorer gross and fine motor functions compared to age-matched, typically developing peers (5). During chemotherapy, patients experience significant declines in quadriceps strength, functional mobility, and cardiorespiratory endurance, reaching a nadir following induction therapy; this underscores the critical importance of early rehabilitation interventions (6). As a safe and effective non-pharmacological approach, exercise intervention is highly regarded in pediatric oncology rehabilitation. Supervised hospital-based exercise programs are not only safe but also provide short-term cardiopulmonary protection and help reduce the length of hospital stays, thereby alleviating the economic burden on families (7). However, in clinical practice, exercise among children with leukemia during treatment is generally characterized by low volume, low intensity, short duration, and limited variety. A lack of systematic physical exercise may lead to a continuous deterioration of motor skills during the treatment period (8).

Currently, while international systematic research on pediatric AL is increasing, a scientific and reliable standardized exercise intervention protocol has yet to be established. In clinical practice, multidisciplinary teams still lack unified evidence-based guidance regarding the timing of interventions and the formulation of stage-specific exercise plans. Therefore, this study utilizes a Best Evidence Summary (BES) design to systematically retrieve and synthesize relevant global literature, aiming to provide a scientific reference for multidisciplinary teams to implement standardized clinical rehabilitation practices.

## 2 Methods

This review was prospectively registered in the International Prospective Register of Systematic Reviews (PROSPERO; registration number: CRD420261341113).

### 2.1 Study design

This study was designed as a Best Evidence Summary based on the JBI methodology for evidence-based practice. A Best Evidence Summary aims to extract actionable clinical recommendations by integrating pre-appraised, high-level evidence. According to the “6S” evidence pyramid model, clinical guidelines and systematic reviews were combined in this study as they represent the most robust sources (summaries and syntheses) to guide clinical decision-making for frontline healthcare professionals. The reporting of this study's search and screening process was guided by the PRISMA statement.

### 2.2 Formation of the evidence-based question

The evidence-based question for this study was formulated using the PIPOST tool:

- P (Population): Children and adolescents diagnosed with acute leukemia.
- I (Intervention): Various forms of exercise interventions, including aerobic exercise, resistance training, and combined physical-mental interventions.
- P (Professional): Nursing staff, physical therapists, and members of multidisciplinary teams.
- O (Outcome): Outcome measures including safety, fatigue, bone mineral density, physical function, and quality of life.
- S (Setting): Hospitals, homes, and community settings.
- T (Type of Evidence): Clinical decision supports, guidelines, expert consensuses, systematic reviews, or meta-analyses.

### 2.3 Literature search strategy

Guided by the “6S” evidence pyramid model, a systematic “top-down” search was conducted to identify relevant literature. The search period spanned from the inception of each database to January 2026.

Search Terms:

- English keywords included: “Child/Adolescent/Infant/Pediatrics,” “Leukemia/Acute lymphoblastic leukemia/Acute lymphocytic leukemia/Acute myeloid leukemia/Child cancer/Pediatric oncology/Pediatric tumors,” and “Exercise/Exercise therapy/Rehabilitation/Physical therapy modalities/Physical fitness”.
- Chinese keywords included: “儿童/患儿/青少年”, “急性白血病/急性淋巴细胞白血病/ALL/AML”, “运动/锻炼/体育活动/康复训练/有氧运动”.

Databases Consulted:

- Evidence-based resources and guidelines: UpToDate, BMJ Best Practice, JBI Evidence-Based Practice Database, Guidelines International Network (GIN), National Comprehensive Cancer Network (NCCN), National Institute for Health and Care Excellence (NICE), and Medlive Guidelines.
- English comprehensive databases: PubMed, The Cochrane Library, Embase, Web of Science, and CINAHL.
- Chinese comprehensive databases: China National Knowledge Infrastructure (CNKI), Wanfang Database, VIP Database, and China Biology Medicine disc (CBM).
- Additionally, a manual search of the reference lists of the included articles was performed (backward citation tracking) to ensure a comprehensive acquisition of relevant literature.

### 2.4 Inclusion and exclusion criteria

#### 2.4.1 Inclusion criteria

The inclusion criteria were as follows:

- Population: Children or adolescents (aged 0–18 years) with a confirmed diagnosis of acute leukemia.
- Interventions: Any form of physical activity or exercise intervention.
- Study Design: Clinical decision supports, guidelines, expert consensuses, systematic reviews, or meta-analyses.
- Language: Publications restricted to Chinese or English.

#### 2.4.2 Exclusion criteria

Literature was excluded if it met any of the following conditions:

- The full text was unavailable.
- The publication was an outdated version of updated literature.
- The study was a duplicate publication.
- The literature was of poor quality upon methodological assessment.

### 2.5 Literature screening

The retrieved literature was imported into EndNote 21 reference management software to identify and remove duplicate records. Two researchers, both systematically trained in evidence-based medicine, independently performed the literature screening based on the predefined inclusion and exclusion criteria. The initial screening involved reviewing titles and abstracts to exclude studies that were clearly irrelevant or did not align with the research design. Subsequently, a full-text review was conducted for the remaining articles during the secondary screening stage to determine the final inclusion. Upon completion of the screening process, the two researchers cross-checked their results. Any discrepancies were resolved through discussion or by consulting a third expert to reach a consensus

### 2.6 Quality appraisal of the literature

Two researchers, both systematically trained in evidence-based medicine, independently evaluated the quality of the included guidelines and expert consensuses using the Appraisal of Guidelines for Research and Evaluation II (AGREE II) instrument (9). This tool comprises 23 items across six domains: scope and purpose, stakeholder involvement, rigor of development, clarity of presentation, applicability, and editorial independence. Each item is scored on a scale from 1 to 7. The standardized percentage score for each domain was calculated as follows:

$$\mathrm{Standardized}\;\mathrm{Domain}\;\mathrm{Score}=\frac{\mathrm{Obtained}\;\mathrm{Score}-\mathrm{Minimum}\;\mathrm{Possible}\;\mathrm{Score}}{\mathrm{Maximum}\;\mathrm{Possible}\;\mathrm{Score}-\mathrm{Minimum}\;\mathrm{Possible}\;\mathrm{Score}}\times 100\text{\%}$$

Based on these domain scores, guidelines were categorized into three grades: A (Strongly Recommended), B (Weakly Recommended), and C (Not Recommended).

For systematic reviews and meta-analyses, the Joanna Briggs Institute (JBI) Critical Appraisal Tool (2016 edition) was utilized (10). This checklist consists of 11 items, with response options of “Yes,” “No,” “Unclear,” or “Not Applicable”.

### 2.7 Data extraction and synthesis

Two researchers, both systematically trained in evidence-based medicine, performed data extraction from the included literature. The extracted information encompassed the year of publication, country of origin, study design, target population, specific intervention details (exercise frequency, intensity, time, and type), outcome measures, and primary conclusions.

Evidence was graded using the Joanna Briggs Institute (JBI) Evidence Levels (2014 edition) (10). The recommendation levels—Grade A (Strong Recommendation) or Grade B (Weak Recommendation)—were determined based on a comprehensive assessment of the evidence's feasibility, appropriateness, meaningfulness, and effectiveness (FAME).

To ensure the precise applicability of the evidence, the specific target populations of the source literature were strictly evaluated. Evidence derived exclusively from acute leukemia (AL) cohorts was classified as direct evidence. Evidence derived from broader pediatric oncology populations or mixed cancer cohorts was classified as indirect evidence. In accordance with the JBI FAME framework, extrapolating findings from mixed populations to AL patients compromises the “Appropriateness” and “Feasibility” components due to differing treatment toxicities. Consequently, the strength of recommendations for all indirect evidence items was systematically downgraded. Every synthesized evidence item is explicitly labeled as direct or indirect to ensure complete transparency for clinical end-users.

Furthermore, a targeted assessment of the primary studies within the included systematic reviews revealed substantial principal overlap. A core group of foundational primary trials was repeatedly cited across multiple reviews, which could artificially inflate the perceived consistency of the evidence. This structural overlap was explicitly factored into the evidence grading process. The recognition that some highly consistent conclusions were driven by the repeated inclusion of the same trials, rather than genuinely independent evidence, served as an additional justification for adopting a conservative grading approach and downgrading the recommendation strengths where appropriate.

## 3 Results

### 3.1 Literature search results

A total of 1,550 records were initially identified through systematic searches across major guideline websites and electronic databases. After removing duplicates and conducting an initial screening of titles and abstracts, 33 articles remained for full-text eligibility assessment. Following a rigorous secondary screening process, 18 moderate-to-high quality publications were ultimately included for evidence synthesis. The detailed selection process is illustrated in the PRISMA flow diagram (Figure 1).

**Figure 1:**
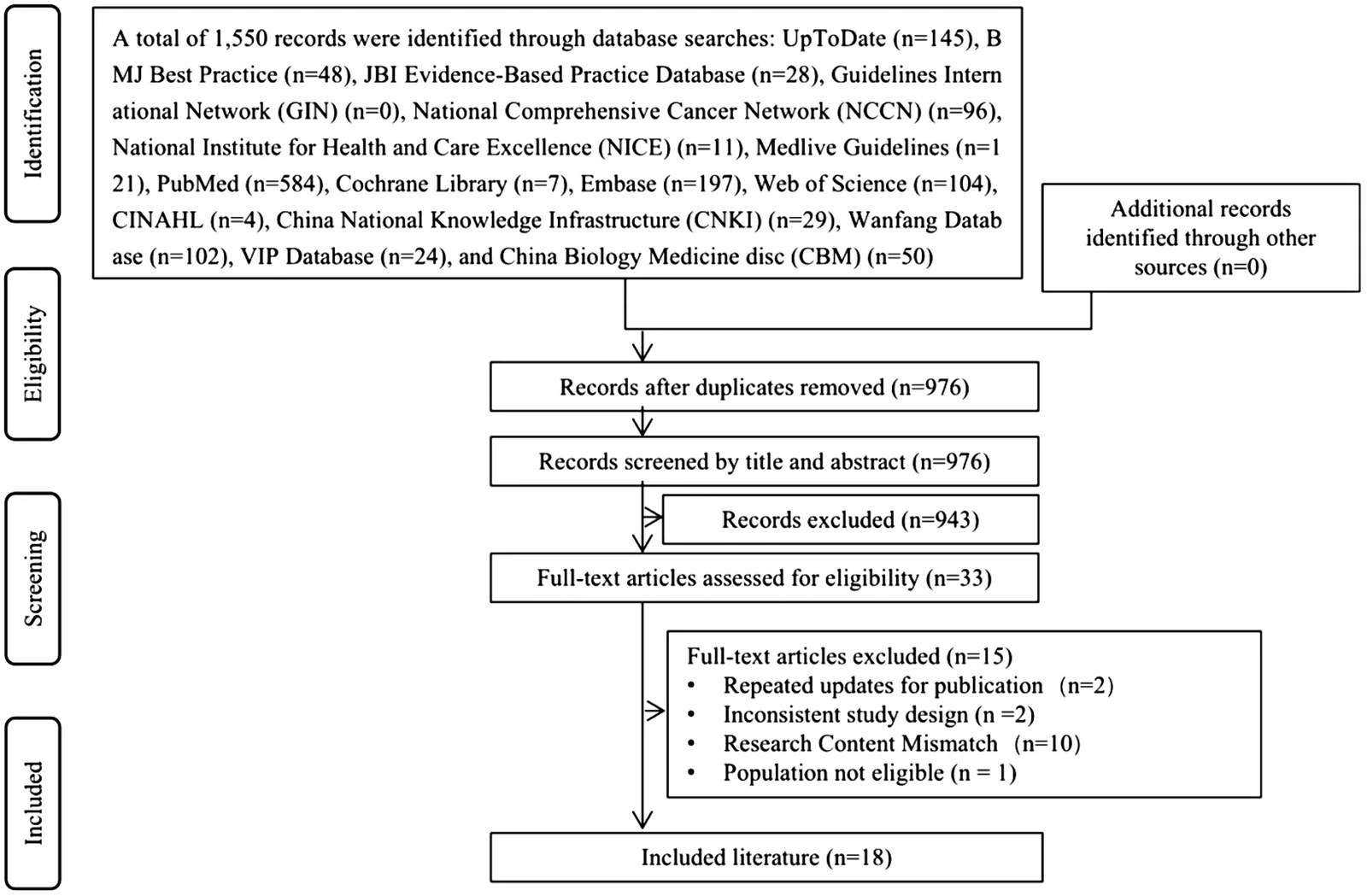
Flow chart of literature screening.

### 3.2 Basic characteristics of the included literature

The 18 included publications comprised 2 clinical practice guidelines and 16 systematic reviews. The key characteristics of these studies, including the year of publication, country of origin, research topic, and intervention details, are summarized in Table 1.

**Table 1:** Basic characteristics of the included literature.

Author (Year)	Source database	Study design	Research topic
Baumann et al. (2013) (11)	PubMed	Systematic Review	Clinical exercise interventions in pediatric oncology
Beller et al. (2021) (12)	PubMed	Systematic Review	Effects of exercise interventions on immune function in children and adolescents with cancer and hsct recipients
Bilazer et al. (2025) (13)	PubMed	Systematic Review	Effectiveness of home-based exercise programs in children with acute lymphoblastic leukemia
Braam et al. (2013) (14)	PubMed	Systematic Review	Physical exercise training interventions for children and young adults during and after treatment for childhood cancer
Brito-Suárez et al. (2022) (15)	PubMed	Systematic Review	Gross motor disorders in pediatric patients with acute lymphoblastic leukemia and survivors
Chang et al. (2013) (16)	PubMed	Systematic Review	Nonpharmacological interventions for fatigue in children and adolescents with cancer
Coombs et al. (2020) (17)	PubMed	Systematic Review	The effect of exercise and motor interventions on physical activity and motor outcomes during and after medical intervention for children and adolescents with acute lymphoblastic leukemia
L'Hotta et al. (2023) (18)	PubMed	Systematic Review	Clinical practice guideline and expert consensus recommendations for rehabilitation among children with cancer
Liu et al. (2024) (19)	PubMed	Systematic Review	Efficacy of physical exercise intervention on children with acute lymphoblastic leukemia during treatment and rehabilitation
Malysse et al. (2021) (20)	PubMed	Systematic Review	Physical Activity Promotion Programmes in Childhood Cancer Patients and Their Impact on Fatigue and Pain
Morales et al. (2018) (21)	PubMed	Systematic Review	Exercise training in childhood cancer
Morales et al. (2021) (7)	PubMed	Systematic Review	Exercise and Childhood Cancer
Patel et al. (2023) (22)	Web of Science	Guideline	Guideline for the management of fatigue in children and adolescents with cancer or pediatric hematopoietic cell transplant recipients
Santos et al. (2020) (23)	PubMed	Systematic Review	Effects of physical exercise during hospitalization in children and adolescents with cancer
Vasconcelos and Sousa (2024) (24)	PubMed	Systematic Review	Effects of Combined Interventions of Physical Activity and Diet in Childhood Cancer Survivors
Wurz and Brunet (2016) (25)	PubMed	Systematic Review	The effects of physical activity on health and quality of life in adolescent cancer survivors
Wurz et al. (2021) (26)	Web of Science	Guideline	The international Pediatric Oncology Exercise Guidelines
Zucchetti et al. (2018) (4)	PubMed	Systematic Review	Exercise program for children and adolescents with leukemia and lymphoma during treatment

### 3.3 Quality appraisal results

#### 3.3.1 Quality appraisal of clinical practice guidelines

A total of two clinical practice guidelines were included in this study, both of which were classified as Grade A (Strongly Recommended) (22, 26). As shown in Table 2.

**Table 2:** AGREE II quality appraisal results for the included guidelines.

Included literature	Scope and purpose	Stakeholder involvement	Rigor of development	Clarity of presentation	Applicability	Editorial independence	Recommendation grade
Patel et al. (2023) (22)	91.7	83.3	100.0	91.7	66.7	91.7	Grade A
Wurz et al. (2021) (26)	91.7	100.0	75.0	66.7	66.7	91.7	Grade A

#### 3.3.2 Quality appraisal of systematic reviews

Among the 16 included systematic reviews and meta-analyses (4, 7, 11–21, 23–25), four studies—Baumann et al. (11), Liu et al. (19), Morales et al. (21), and Vasconcelos and Sousa (24)—met all 11 criteria of the JBI Critical Appraisal Tool. Detailed appraisal results are summarized in Table 3. No reviews were found to have fatal methodological flaws that warranted exclusion. However, the specific methodological limitations present in these 12 reviews were actively factored into the subsequent evidence grading process. These shortcomings reduced the “Appropriateness” and “Feasibility” components within the JBI FAME framework, serving as a direct rationale for systematically downgrading the strength of recommendations derived from these reviews in our best evidence summary.

**Table 3:** Methodological quality appraisal of the included systematic reviews (*n* = 16).

Author (Year)	Q1	Q2	Q3	Q4	Q5	Q6	Q7	Q8	Q9	Q10	Q11
Baumann et al. (2013) (11)	Y	Y	Y	Y	Y	Y	Y	Y	Y	Y	Y
Beller et al. (2021) (12)	Y	Y	Y	Y	Y	Y	Y	Y	U	Y	Y
Bilazer et al. (2025) (13)	Y	Y	Y	Y	Y	Y	Y	N/A	N/A	Y	Y
Braam et al. (2013) (14)	Y	Y	Y	Y	Y	Y	Y	Y	U	Y	Y
Brito-Suárez et al. (2022) (15)	Y	Y	Y	Y	Y	Y	Y	Y	U	Y	Y
Chang et al. (2013) (16)	Y	Y	Y	Y	Y	Y	Y	Y	U	Y	Y
Coombs et al. (2020) (17)	Y	Y	Y	Y	Y	Y	Y	Y	U	Y	Y
L'Hotta et al. (2023) (18)	Y	Y	Y	Y	Y	N	Y	Y	U	Y	Y
Liu et al. (2024) (19)	Y	Y	Y	Y	Y	Y	Y	Y	Y	Y	Y
Malysse et al. (2021) (20)	Y	Y	Y	Y	Y	Y	Y	N/A	N/A	Y	Y
Morales et al. (2018) (21)	Y	Y	Y	Y	Y	Y	Y	Y	Y	Y	Y
Morales et al. (2021) (7)	Y	Y	Y	Y	Y	Y	Y	Y	U	Y	Y
Santos et al. (2020) (23)	Y	Y	Y	Y	Y	U	U	Y	U	Y	Y
Vasconcelos and Sousa (2024) (24)	Y	Y	Y	Y	Y	Y	Y	Y	Y	Y	Y
Wurz and Brunet (2016) (25)	Y	Y	Y	Y	N	U	U	N/A	N/A	Y	Y
Zucchetti et al. (2018) (4)	Y	Y	Y	U	Y	Y	Y	Y	N/A	Y	Y

Q1: Was the review question clearly and explicitly stated? Q2: Were the inclusion criteria appropriate for the review question? Q3: Was the search strategy appropriate? Q4: Were the sources searched adequate? Q5: Were the criteria for appraising studies appropriate? Q6: Was critical appraisal conducted by two or more reviewers independently? Q7: Were there strategies to minimize errors in data extraction? Q8: Were the methods used to combine studies appropriate? Q9: Was the likelihood of publication bias assessed? Q10: Were recommendations for policy and/or practice supported by the reported data? Q11: Were the specific directives for new research appropriate? Y, Yes; N, No; U, Unclear; NA, Not Applicable.

### 3.4 Summary of best evidence for exercise intervention in children with acute leukemia

A total of 17 best evidence items were synthesized, categorized into eight dimensions: intervention principles, safety, exercise modalities, intensity, frequency and timing, stage-specific strategies, intervention settings, and outcome improvement. The summary of evidence is presented in Table 4.

**Table 4:** Summary of best evidence for exercise intervention in children with acute leukemia.

Category	Evidence content	Level of evidence	Grade of recommendation
Intervention principles	1) [Indirect Evidence: Mixed Pediatric Oncology] Physical activity and exercise are generally safe and beneficial for the vast majority of clinically stable children and adolescents affected by cancer; “staying active” throughout the entire treatment process should be encouraged (7, 26).	Level 5.b	B
	2) [Direct Evidence: AL-specific] Gross motor skill impairments in children with acute lymphoblastic leukemia (ALL) are long-term issues that may persist for 5–6 years post-treatment; therefore, interventions must be a continuous, multi-year rehabilitation process (15).	Level 3.a	B
Safety	1) [Indirect Evidence: Mixed Pediatric Oncology] Implementing exercise interventions during chemotherapy is generally feasible and safe, provided that the program is carefully tailored to the patient's dynamic clinical status. Under strict professional supervision and individualized intensity adjustment, severe adverse events (e.g., fractures or serious cardiovascular events) are rarely reported, though minor events like transient muscle soreness may occur (11, 25).	Level 1.b	B
	2) [Indirect Evidence: Mixed Pediatric Oncology] Exercise interventions do not suppress the vulnerable immune systems of pediatric patients (e.g., no reduction in WBC, neutrophil, or platelet counts). Conversely, moderate exercise may enhance natural killer (NK) cell cytotoxicity; however, direct evidence linking this biological change to clinical outcomes, such as reduced infection risk or shorter hospital stays, remains limited (12).	Level 1.b	B
Exercise modalities	1) [Indirect Evidence: Mixed Pediatric Oncology] As single-mode interventions have limited efficacy, a multimodal exercise program is recommended, including aerobic exercise (e.g., treadmill walking, cycle ergometry) for cardiorespiratory fitness, resistance training (e.g., elastic bands, light dumbbells, or body weight) to counteract muscle atrophy, and flexibility training (stretching) to maintain range of motion (11, 23).	Level 1.b	B
	2) [Indirect Evidence: Mixed Pediatric Oncology] For cancer-related fatigue (CRF), physical activity alone may be insufficient; combining it with psychological interventions, such as relaxation techniques and mindfulness training, can more effectively alleviate physical and mental exhaustion (22).	Level 1.a	B
	3) [Indirect Evidence: Mixed Pediatric Oncology] Utilizing game-based exercise forms (e.g., tossing games, obstacle courses, exergaming) is recommended to enhance patient engagement and improve emotional experiences (23).	Level 1.b	B
Intensity, frequency, and timing	1) [Indirect Evidence: Mixed Pediatric Oncology] Individualized FITT protocols (e.g., 2–3 moderate sessions/week, progressing duration) require dynamic adjustments. Strict termination criteria (e.g., fever >38 °C, severe thrombocytopenia) must mandate exercise withholding based on the patient's acute clinical status and treatment phase (25, 26).	Level 5.b	B
	2) [Indirect Evidence: Mixed Pediatric Oncology] To ensure the safety and efficacy of the intervention, training should be conducted under the guidance of professionals with expertise in pediatric oncology (25).	Level 5.b	B
	3) [Direct Evidence: AL-specific] Early-initiated exercise interventions (within one year of starting treatment) are more effective in protecting bone mineral density (BMD); it is recommended to introduce exercise early after treatment initiation to seize the “golden window” for preventing bone loss (19).	Level 1.a	B
Stage-specific strategies	1) [Direct Evidence: AL-specific] During induction remission (predominantly reflecting ALL protocols), patients are physically weak; exercise should focus on alleviating fatigue and maintaining basic activities of daily living (ADL) rather than high-intensity improvements—requiring extreme caution for AML (17).	Level 1.b	B
	2) [Direct Evidence: AL-specific] Upon entering the maintenance treatment phase, when the condition is relatively stable, the intervention goals should expand to comprehensively improving physical fitness, focusing on increasing range of motion (ROM), enhancing muscle strength, improving BMD, and boosting aerobic capacity to prepare for reintegration into society (17).	Level 1.b	B
Intervention settings	1) [Indirect Evidence: Mixed Pediatric Oncology] During hospitalization (even during isolation in laminar flow rooms), it is generally practical to utilize limited space for bedside strength training, indoor walking, and stretching (23).	Level 1.b	B
	2) [Direct Evidence: AL-specific] For the long-term maintenance phase, home-based exercise programs are a reliable training model; home-based exercise implemented via remote guidance or regular follow-ups demonstrates acceptable feasibility and significantly improves health biomarkers in children (13).	Level 1.b	B
Outcome improvement	1) [Indirect Evidence: Mixed Pediatric Oncology] Physical activity significantly reduces systemic fatigue in children with cancer. Compared to pharmacological or other therapies, exercise intervention has been established as the preferred non-pharmacological approach for managing cancer-related fatigue (CRF) (16, 20, 22).	Level 1.a	B
	2) [Indirect Evidence: Mixed Pediatric Oncology] Chemotherapy drugs (e.g., glucocorticoids, methotrexate) can cause severe bone loss; regular exercise intervention can effectively maintain or improve systemic BMD in pediatric patients (14, 19, 25).	Level 1.a	B
	3) [Direct Evidence: AL-specific] Long-term, regular exercise intervention yields broad physiological benefits, significantly improving cardiorespiratory endurance (e.g., 9-min run/walk performance), lower limb and back muscle strength, and overall quality of life (QoL) in children with ALL (14, 19, 21).	Level 1.a	B

## 4 Discussion

Based on the “6S” evidence pyramid model, this study systematically synthesized 17 best evidence items regarding exercise interventions for children with acute leukemia (AL), encompassing eight core dimensions: intervention principles, safety, exercise modalities, intensity, frequency and timing, stage-specific strategies, intervention settings, and outcome improvement. It is imperative to clarify that these 17 evidence items and their assigned recommendation grades were derived strictly and exclusively from the 18 formally appraised secondary studies (clinical guidelines and systematic reviews). To better contextualize these findings, explain potential physiological mechanisms, and offer specific examples of clinical implementation, additional primary studies (e.g., individual clinical trials or observational studies) have been cited throughout this discussion. These supplementary publications serve purely as contextual references and were not part of the predefined JBI evidence grading process. Traditional clinical nursing and home care perspectives often advocate for “absolute bed rest” to conserve energy and prevent complications such as infection or bleeding (27). However, the evidence from this study suggests a necessary paradigm shift: physical activity and exercise are generally safe and beneficial for the vast majority of clinically stable children and adolescents affected by cancer. Consequently, “staying active” should be encouraged throughout the entire treatment continuum (7, 26, 28).When implemented under rigorous assessment and professional supervision, exercise programs rarely lead to fractures or serious cardiovascular events (11, 25, 28). According to the synthesized evidence, the most frequently reported adverse events are typically mild and self-limiting, including transient fatigue, delayed-onset muscle soreness, and minor joint pain. However, it is imperative to note that the absence of reported severe adverse events in some studies must not be misinterpreted as absolute proof of safety. The overall safety of an exercise program is highly conditional; it relies on meticulous patient selection, continuous monitoring of clinical status (e.g., fluctuating platelet counts or anemia), appropriate exercise intensity, and treatment-related complications. Crucially, exercise intervention does not exert a negative suppressive effect on the fragile immune systems of these children; for instance, it does not reduce white blood cell or platelet counts. On the contrary, moderate exercise can enhance the cytotoxicity of natural killer (NK) cells (12, 29). However, extrapolating this biological enhancement directly to hard clinical outcomes—such as reduced infection risk and shorter hospital stays—requires extreme caution, as the direct causal pathway remains to be fully validated by clinical trials. Despite identifying the general types of adverse events, a major limitation of the current evidence base is the lack of quantitative safety data. It was not possible to quantitatively report the exact number of primary studies that systematically monitored adverse events, the total pool of participants assessed for safety, the precise incidence rates of specific adverse events, or the exact frequency of exercise-related discontinuations. The included secondary source literature—comprising systematic reviews and guidelines—predominantly provided narrative summaries of safety without standardizing adverse-event definitions or conducting quantitative meta-analyses of safety outcomes. Consequently, safety must always be dynamically managed. Therefore, healthcare professionals must transform their clinical approach by introducing exercise interventions early after treatment initiation, once the patient is clinically stable. This proactive strategy is essential to seize the critical “window of opportunity” for preventing complications such as bone loss and should extend into long-term rehabilitation follow-up post-treatment (30).

The cognitive developmental stages and psychological characteristics of children often lead to notoriously poor compliance with traditional, repetitive rehabilitation exercises (31). Evidence from this study indicates that monomodal exercise interventions yield limited efficacy. Instead, a multimodal approach is recommended, encompassing aerobic exercise to enhance cardiorespiratory endurance, resistance training to counteract muscle atrophy, and flexibility training to maintain range of motion (ROM) (11, 23, 32). To improve clinical feasibility and patient adherence, the evidence strongly suggests incorporating game-based exercise formats (33). By integrating monotonous resistance and aerobic tasks into activities such as tossing games, obstacle courses, or interactive exergaming, clinicians can effectively facilitate cognitive distraction from chemotherapy-related side effects. This approach significantly boosts intrinsic motivation and engagement while mitigating negative emotional experiences (23, 34). Furthermore, for severe cancer-related fatigue (CRF), physical intervention alone often falls short of optimal outcomes (22, 35). The evidence recommends combining physical activity with psychological support (22), such as relaxation techniques and mindfulness training. Such multimodal strategies provide comprehensive rehabilitation support by addressing both physiological and psychological dimensions.

The treatment cycle for acute leukemia is prolonged, and the toxicities and adverse effects vary significantly across different stages (36); Individualized FITT parameters (e.g., 2–3 moderate sessions/week) require dynamic adjustments. Crucially, strict termination criteria—including active fever (>38 °C), severe anemia, or profound thrombocytopenia—must dictate the temporary withholding of exercise to ensure safety (25, 37). Furthermore, because the majority of current evidence is derived from acute lymphoblastic leukemia (ALL) cohorts, multidisciplinary teams must exercise additional caution and clinical judgment when applying these interventions to patients with acute myeloid leukemia (AML), who often undergo more intensively myelosuppressive regimens. Evidence suggests that during induction remission (primarily reflecting ALL regimens), high-dose chemotherapy causes extreme weakness and myelosuppression (19, 38). Consequently, the primary objective should focus on alleviating fatigue and maintaining basic activities of daily living (ADL) rather than high-intensity enhancement, with careful protocol-specific adaptations for AML. As patients transition into the maintenance therapy phase, their condition becomes relatively stable. During this period, intervention goals should shift toward comprehensively improving range of motion (ROM), enhancing muscle strength, increasing bone mineral density, and boosting aerobic capacity to prepare the child for reintegration into school and society (39). In terms of setting, utilizing limited hospital space for bedside strength training, indoor walking, and stretching is generally practical during inpatient stays (40). Once patients enter the maintenance phase or are discharged, home-based exercise programs—implemented through remote guidance, wearable health monitoring devices, or regular outpatient follow-ups—demonstrate acceptable feasibility. These programs not only significantly improve various health biomarkers but also alleviate the time and economic burden associated with frequent hospital visits (13, 41).

## 5 Conclusion and limitations

### 5.1 Conclusion

Exercise intervention for children with acute leukemia should adhere to the fundamental principles of being long-term, continuous, and individualized, and must be conducted under professional supervision. Interventions should span the entire treatment continuum, utilizing a multimodal approach that integrates aerobic, resistance, and flexibility training with psychological adjustment. Provided that safety is rigorously ensured, exercise contributes to potentially supporting immune function, alleviating cancer-related fatigue (CRF), maintaining or improving bone mineral density (BMD), and enhancing muscle strength, cardiorespiratory endurance, and overall quality of life. Implementation strategies must be dynamically adjusted according to the treatment phase, and the use of game-based formats is encouraged to improve adherence. In summary, while current evidence limitations (such as indirectness and overlapping data) warrant careful clinical application, systematic and scientific exercise intervention constitutes a highly valuable and promising non-pharmacological component of comprehensive supportive care for pediatric patients with acute leukemia, though specific adaptations for non-ALL subtypes, such as AML, require further empirical validation.

### 5.2 Limitations

This study has several limitations that warrant acknowledgment. First, the synthesized evidence predominantly focuses on children with acute lymphoblastic leukemia (ALL), while exercise intervention strategies for other subtypes, such as acute myeloid leukemia (AML), remain relatively scarce. Second, significant heterogeneity exists across secondary sources regarding populations, interventions, and measurement tools. Consequently, the volume, quality, and directness of evidence vary considerably among specific outcomes (e.g., fatigue versus immune function), meaning the overall degree of clinical certainty is not uniform across all synthesized statements. Third, although we qualitatively synthesized the evidence and identified the principal overlap of foundational trials, a formal structured assessment to quantify the exact degree of overlap—such as generating a comprehensive citation matrix or calculating the Corrected Covered Area (CCA)—was not performed due to the scope of this secondary synthesis. Consequently, it remains a limitation that readers cannot fully determine whether all apparently consistent conclusions reflect genuinely independent evidence or the repeated inclusion of the same primary trials. Fourth, some extracted recommendations are derived from evidence based on mixed pediatric oncology populations rather than strictly AL cohorts. Because many of these source reviews did not report specific sub-population data, we were unable to quantify the exact proportion of AL patients within this indirect evidence. Finally, the reliance on secondary literature prevented a quantitative synthesis of safety metrics. The source systematic reviews largely lacked standardized reporting of precise adverse event frequencies and exercise-related dropout rates, leaving a critical gap in precisely quantifying intervention risks. Future research should prioritize multicenter clinical trials to further validate and refine the evidence summarized in this study.

## Data Availability

The original contributions presented in the study are included in the article/Supplementary Material, further inquiries can be directed to the corresponding authors.
